# Improvement of mechanical properties of elastic materials by chemical methods

**DOI:** 10.1080/14686996.2020.1849931

**Published:** 2021-02-01

**Authors:** Yukikazu Takeoka, Sizhe Liu, Fumio Asai

**Affiliations:** a Department of Molecular and Macromolecular Chemistry, Graduate School of Engineering, Nagoya University, Nagoya, Japan; b Research & Development Center, Kyoto, Japan

**Keywords:** Elastomer, gel, mechanical property, crosslink, polyrotaxane, 101 Self-assembly / Self-organized materials, 303 Mechanical / Physical processing

## Abstract

Elastomers such as gels and rubbers play various roles in our lives. Elastomers, which guarantee the safety of airplanes and automobiles and the stability of buildings, are materials that have made the lives of people in the twentieth century extremely convenient. The existence of macromolecules, that is, giant molecules, has been clarified; the development of synthetic macromolecules has progressed; and understanding of elastomers has progressed. By introducing new ideas, it has become possible to obtain tough and hard elastomers, which was difficult under conventional ideas. In this paper, we will explain the development from the classical theory of elastomers to current efforts.

## Graphical Abstract

**Figure:**
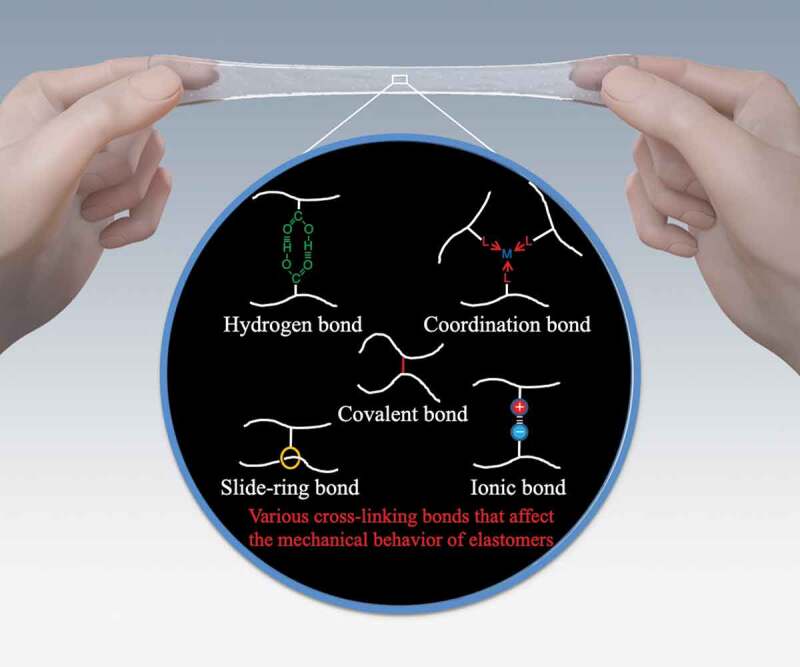

## 1. Introduction

It has been 100 years since Germany’s Prof. Staudinger proposed the existence of a type of molecule called a ‘polymer’, which is a macromolecule linked by chemical bonds, in 1920[1]. In the twentieth century, synthetic polymers made great industrial progress, and polymers are said to be the most versatile and convenient materials invented by mankind. However, it has been a problem to improve the polymer property of becoming fragile when made thin and brittle when made thick and hard. In Japan, for five years starting in 2014, research groups from companies, universities, and national research institutes participated in ImPACT, an innovative research and development promotion program under the program manager Prof. Kozo Ito of the University of Tokyo, to pursue ‘The realization of ultra-thin film and toughening flexible polymer’[2]. In this project, they researched the toughness of soft materials with the goal of realizing a flexible tough polymer that simultaneously achieves thinning and toughness that exceed conventional limits.

Soft elastic materials such as gel and rubber are generally called elastomers. In our daily lives, the mechanical properties of elastomers are useful in various applications, such as automobile and airplane parts, vibration-proof materials for precision machines and buildings, and sports equipment. High-performance elastomers are indispensable for important industries that support our future lives, such as advanced medicine and soft robots.

**Figure 1.**
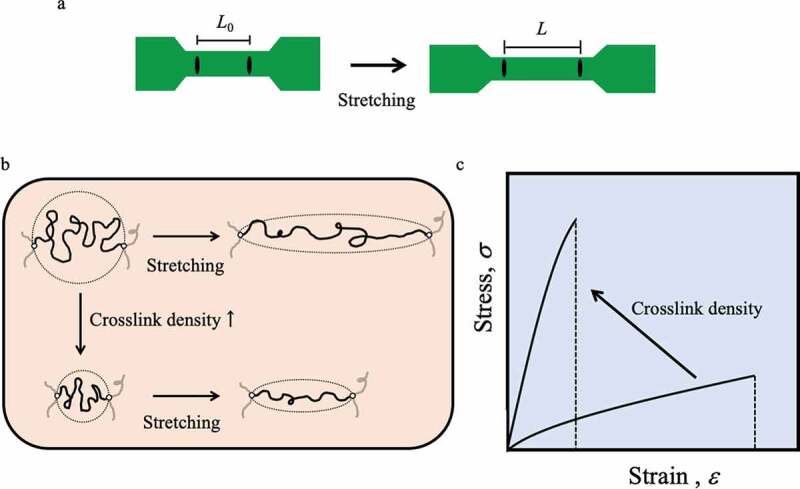
(a) Strain evaluation when the dumbbell-shaped sample is uniaxially stretched: the strain value *ε* is measured by setting the initial value *L*_0_ as the length between the marked lines and measuring the length *L* between the marked lines as the sample stretches: *ε* (%) (=100 × (*L* − *L*_0_)/*L*_0_). (b) Conceptual diagram of the extension of partial chains when uniaxially extending networks with different molecular weights of partial chains between crosslinking points by changing crosslink density. (c) A conceptual diagram showing that the elastic modulus and extensibility on the stress–strain curve change when an elastomer is prepared with different crosslink density

The most basic evaluation of the mechanical properties of elastomers is the stress-strain property, which is evaluated by standardizing the force and the amount of deformation to the force (stress, *σ*) applied to the unit area and the ratio of the amount of deformation to the original dimension (strain, *ε*) so as not to depend on the shape and size of the elastomer sample[3]. Therefore, the mechanical characteristics peculiar to the material can be known. The tensile test is the most frequently used method for testing the relationship between stress and strain. A dumbbell-shaped sample, as shown in Figure 1(a), is cut out; both ends are attached to a tester; and the relationship between stress and strain generated by uniaxial stretching is measured. Marks are made at two points on the thin part of the dumbbell-shaped sample with a marker, and the change in length between the marked lines is measured to obtain the strain. The strain value *ε* (%) is calculated from 100 × (*L* − *L*_0_)/*L*_0_, where *L*_0_ is the length between the marked lines before stretching and *L* is the length after stretching. If the cross-sectional area of the thin part of the dumbbell-shaped sample before stretching is *A*_0_, the stress *σ* is the value obtained by dividing the tension *f* generated during sample stretching by *A*_0_. The elastomer undergoes a large deformation at low tension and returns to its original shape when tension is released. For an elastomer to exhibit such a response, it must have sufficient molecular mobility to enable distortion of the conformation of the polymer under the force applied and crosslinking between the polymers to prevent flow and provide restoring force (Figure 1(b)). The elongation until breakage of the crosslinked elastomer is proportional to one half of the molecular weight between crosslink points of the polymer chains constituting the elastomer. Therefore, even for the same material, the mechanical properties of the elastomer can be adjusted simply by changing the crosslink density. However, when the crosslink density is increased to increase the elastic modulus, the elongation is rapidly impaired (Figure 1(c)). Generally, the extensibility and elasticity of a conventional elastomer with a crosslinked structure are in a trade-off relationship, and it is not easy to improve the toughness of the elastomer.

To make human life richer and more convenient in the future, it will be necessary to develop elastomers that exhibit mechanical properties according to their use. Many years of research have clarified the relationship between the molecular structure that composes the elastomer constructed by conventional methods and its mechanical properties. The development of elastomers based on this idea has become important. In recent research, various methods, such as the introduction of a new crosslinked structure [4] and the formation of a uniform network structure[5], have been studied as methods for increasing the toughness of an elastomer. In this paper, we introduce the kinds of efforts being made from a molecular perspective to improve the mechanical properties of elastomers such as gels.

## 2. Uniaxial stretching of one polymer chain for toughening of soft materials

The elasticity that occurs when a spring made of metal or the like is stretched is caused by the distance between the atoms that make up the spring being increased by stretching. That is, the spring tries to contract due to energy elasticity. How can we explain the expansion and contraction of gels composed of polymer chains and elastomers such as rubber? First, let us consider the single polymer chain that constitutes the elastomer[6].

**Figure 2.**
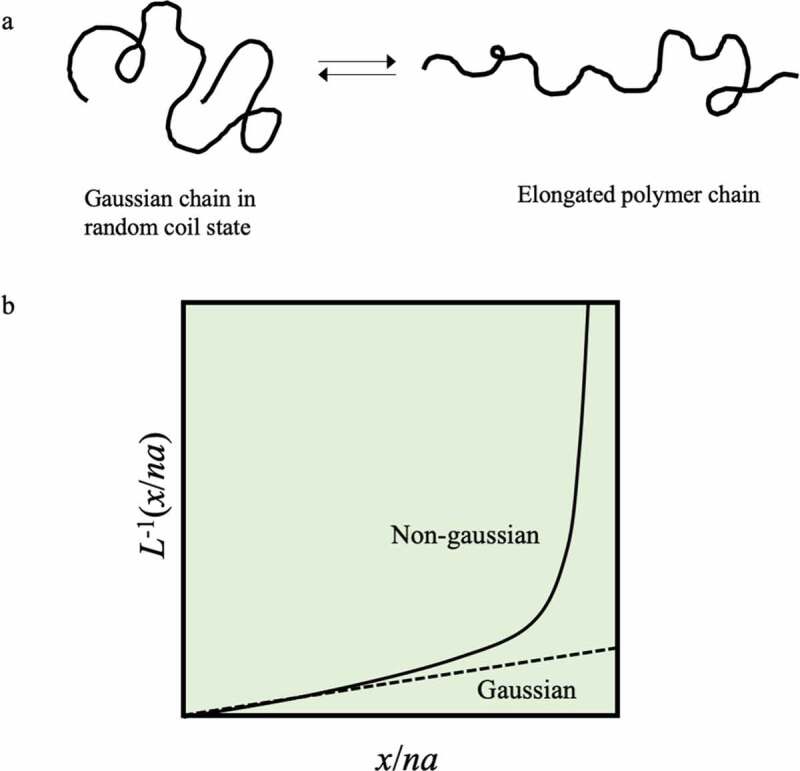
(a) Conformational changes between a Gaussian state and an extended state of an isolated polymer chain. (b) Change in the conformation of a single polymer chain according to Gaussian chain theory (dotted line) and change by the non-Gaussian chain theory explained by applying the inverse Langevin function L^−1^ (solid line)

A general linear polymer chain has a diameter of approximately 0.1 nm, and when the molecular weight is 100,000, the fully extended length is approximately 1000 nm. For example, polyethylene, which is the simplest structure, has a structure in which carbon is connected by covalent bonds in the main chain, and the stable angle of C-C-C is approximately 109.5°. At room temperature, this chain is constantly and drastically changing its conformation due to 1) stretching motions between bond points, 2) bending motions of bond angles, and 3) rotational motion at bond points. However, considering the potential energy associated with the rotation of the C-C bond, it can be in the gauche state or the trans state, so that the entire polymer chain exists in a random coil state in which the string is rolled. Judging from the fact that the conformation is changed by thermal motion, it will be possible to easily stretch with a relatively weak force by grasping both ends of this polymer chain and stretching it in one direction. At this time, energy elasticity occurs in phenomena such as 1) and 2) that change the distance between atoms, but these phenomena can be ignored, and the C-C bond rotation in 3) is the most important. The chain is thereby extended. Nevertheless, the polymer chains try to return to their original random coil state, just as when the spring is stretched. This is due to the entropic elasticity of the stretched chains. When the distance *x* between the ends of the polymer chain follows a Gaussian distribution, the polymer chain with such characteristics is called a Gaussian chain. In its natural state, the polymer chain is a Gaussian random coil, and entropy decreases when trying to extend it from this state (Figure 2(a)). Therefore, entropy elasticity is generated by the propensity to return to the original state. In other words, the elasticity of a rubber made of a polymer chain and of a spring made of a metal is based on different principles. It is known that the tension *f* generated by the entropy elasticity of a single chain is expressed by the following equation in terms of statistical mechanics.

(1) $$f=\frac{3k_{B}T}{na^{2}}x$$

Here, *k*_B_ is the Boltzmann constant, *T* is the absolute temperature, *n* is the number of monomers constituting the polymer, *a* is the size of the monomers, and *x* is the elongation. In other words, it can be seen that entropy elasticity also follows Hooke’s law. However, formula (1) is obeyed until approximately one-third of the length until the polymer chain is fully extended, and the change in tension due to further extension cannot be explained by formula (1). This is because if the chain is stretched to such an extent that it will be affected by the stretching, it will be outside the Gaussian distribution.

The relationship between the elongation and tension of polymer chains under large elongation is explained by applying the Langevin function *L* as ‘non-Gaussian chain theory’ [3,7]:

(2). $$f=\left(\frac{kT}{a}\right)L^{-1}\left(\frac{x}{na}\right)$$

Expanding this formula for *x*/*na*, we can obtain the following equation:

(3). $$\begin{array}{rl} & f=\left(3kT\frac{x}{na^{2}}\right)\left[1+\left(\frac{3}{5}\right){\left(\frac{x}{na}\right)}^{2}+\left(\frac{99}{175}\right){\left(\frac{x}{na}\right)}^{4}\right.\\ & \;\;\;\;\;\;\;\;\;\;\;\;\left.+\left(\frac{513}{875}\right){\left(\frac{x}{na}\right)}^{6}+\cdot \cdot \cdot \right] \end{array}$$

The difference from the relationship between tension and elongation expressed by equation (1) is shown in Figure 2(b). The change indicated by the solid line obtained from equation (3) shows the change in tension due to the extension of the chain, resulting in a fully extended state. This model was applied by Kuhn and Grün because the relationship between applied stress and segment orientation is similar to the relationship between applied voltage and dipole orientation.

**Figure 3.**
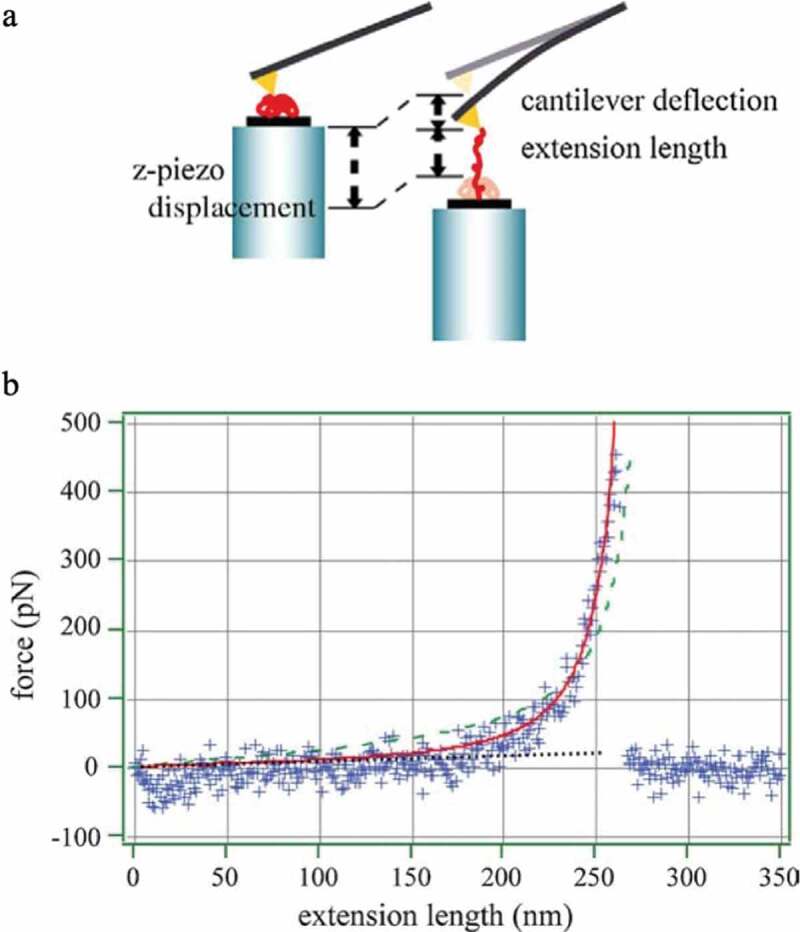
(a) Conceptual diagram for the measurement of mechanical properties of single chains by atomic force microscopy. (b) Tension and extension length results of a single polystyrene chain in cyclohexane at 35°C obtained by the method shown in Figure 3a (reproduced with permission from Elsevier)

Nakajima et al. reported the results of experimental investigations of changes in tension with the extension of a single polymer chain using an atomic force microscope (Figure 3). [8]The distance at which the bond breaks occur is 261 nm, and it can be extended to approximately 90% of the extended chain length of 284.5 nm. The tension–elongation relationship does not change monotonically, as shown in equation (1); instead, the tension increases significantly as the elongation increases, as in the theoretical result shown in Figure 2(b). From this experiment, it can be seen that the assumption of a Gaussian chain, which extends linearly indefinitely, does not hold when the polymer chain is greatly stretched.

## 3. Classical rubber elasticity theory: entropy elasticity

Assuming a Gaussian chain, the tension *f* generated when one polymer chain is stretched is proportional to the elongation *x*, so it was explained in the previous section that the polymer chain can be explained by the Hooke’s equation, similar to a spring. On the other hand, in the extension of a crosslinked body made up of multiple Gaussian chains that are not entangled with polymer chains, it is assumed that the deformation of the partial chains between the crosslinking points is similar to the deformation of the entire material. Based on the rubber elasticity theory described above, the relationship between the extension ratio *λ* and the tension *f* is as follows.

(4) $$f=\frac{vk_{B}T}{V}A_{0}\left(\lambda -\frac{1}{\lambda^{2}}\right)$$

**Figure 4.**
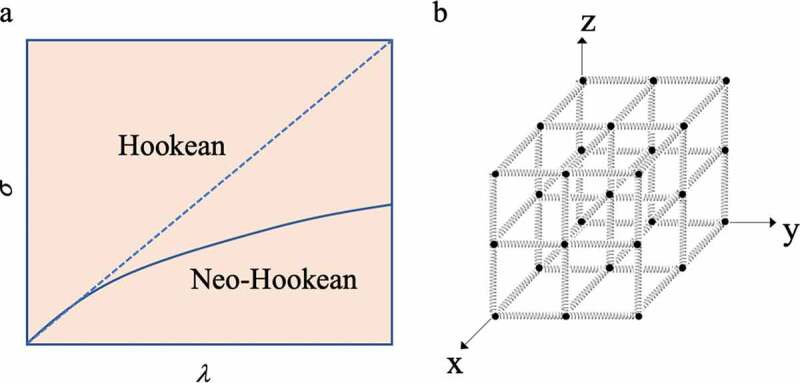
(a) Diagram of the relationship between the stress *σ* and stretch ratio *λ* of the elastomer according to Hooke’s law (dotted line) and of Neo-Hooke’s law (solid line). (b) Conceptual diagram of a network structure in which polymer chains exhibiting Hookean elasticity are connected to form a polymer network in the x, y, and z directions

Here, *ν*/*V* is the number of partial chains (*ν*) per unit volume (*V*), *k*_B_ is the Boltzmann constant, *T* is the absolute temperature, and *A*_0_ is the area of the portion where the force acts during extension of the crosslinked body. *λ* is the ratio (*L*/*L*_0_) of the initial length *L*_0_ before the crosslinked body is stretched and the length *L* when deformed. In other words, *σ* and *λ* of a crosslinked structure composed of multiple Gaussian chains do not have a simple proportional relationship. Since *f*/*A*_0_ is the force per unit area, it is the stress (*σ*) generated in the crosslinked body. As a result, when *σ* is plotted against *λ*, a curve showing a convex shape is obtained (solid line in Figure 4(a)). An elastic body exhibiting such a relationship is called a Neo-Hookean elastic body [3,9,10].

If *N* polymer chains showing Hookean elasticity of *f* = *kx* in the *x*-axis direction are arranged in parallel in an independent state, the total stress generated upon stretching by *λ* times in the *x*-direction becomes *Nkλ*. When the stress and the stretch ratio are in a proportional relationship, it is called a Hookean elastic body and shows a change like the dotted line in Figure 4(a). However, if the polymer chains that exhibit Hookean elasticity are connected to form a network structure in which polymers are arranged in the *x, y*, and *z* directions (Figure 4(b)), if they extend in the *x* direction, contraction occurs in the *y* and *z* directions. This is because there is an assumption that the volume of a material made up of Gaussian chains does not change due to stretching. Therefore, when contraction occurs in the *y*-axis and *z*-axis directions, internal pressure is generated in the network structure. Then, an expansion pressure that resists the internal pressure is also generated. Originally, the elastic force is generated as a stress that resists expansion from the outside, but since expansion pressure is also generated along with the expansion, this serves to partially cancel the stress. As a result, equation (4) is obtained. In other words, this is the result showing that in the three-dimensional network structure, the molecular chains always move in conjunction with each other. This theory is based on the following affine deformation assumptions[11]: 1) polymer chains are long chains of freely rotating bonds; 2) the interaction between chains is negligible; and 3) the deformation of the three-dimensional structure composed of these polymer chains is similar to the deformation of the entire material. Later, Rivlin et al. came to call this theory the classical theory of rubber elasticity, since the difference from the properties of real rubber was pointed out by biaxial stretching experiments[10]. In addition, as in the case of the single-chain polymer chain dealt with in the previous section, when the chain is greatly extended, it is outside the Gaussian distribution, and this theory cannot explain it.

**Figure 5.**
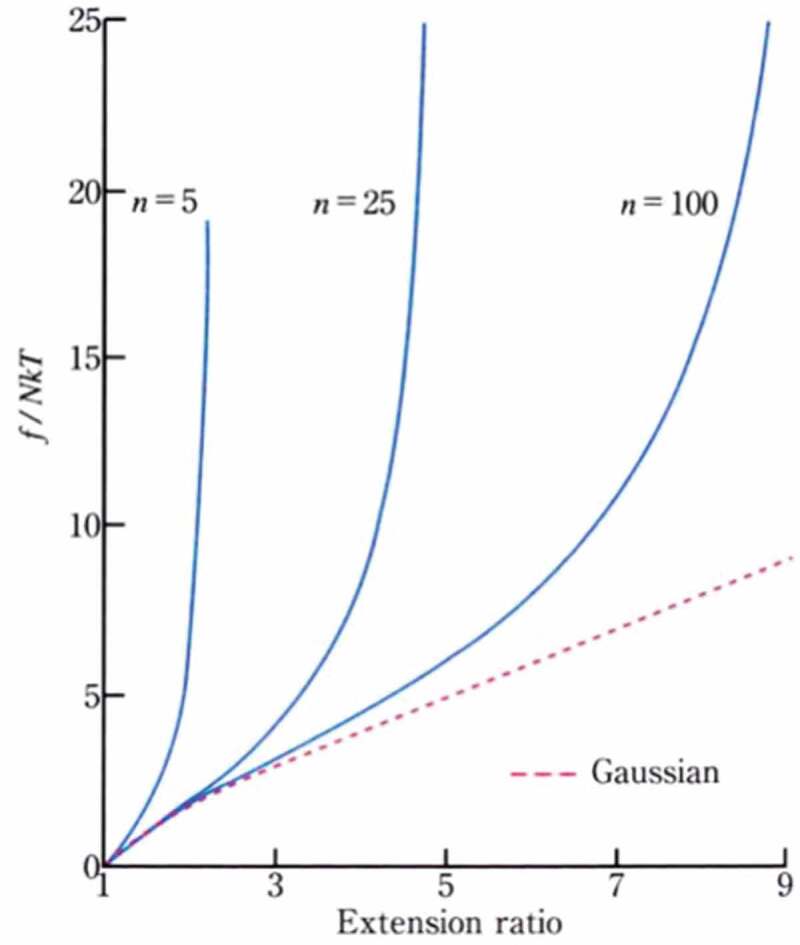
Examples of the entropic elasticity Gaussian chain approximation (dotted line) and non-Gaussian chain approximation (solid line): the example of the non-Gaussian chain approximation also describes the case where the number *n* of monomers constituting the polymer is different (reproduced with permission from Copyright 2009 Physical Society of Japan.)

The treatment by the non-Gaussian chain approximation derived by James and Guth agrees with the Gaussian chain approximation in the region where the stretch ratio is low, but when the stretch ratio increases, the relationship between elongation and tension rises significantly [12] (Figure 5). Additionally, the smaller the number *n* of monomers constituting the polymer is, the earlier the curve rises[13]. However, in an actual system, there are many cases where such a stress-strain change that the polymer chain becomes fully extended is not shown. Since the length of the polymer chains existing between the crosslinking points of the actual network structure has a broad distribution, one of the causes is that the stress is concentrated on the shortest polymer chains under great extension.

## 4. Effect of interaction between polymer chains: energy dissipation (loss) due to viscosity

In the above explanation, the mechanical properties of the elastomer, mainly composed of entropy elasticity, have been explained by ignoring the interactions between the polymer chains constituting the elastomer. For example, in the case of a so-called gel in which a crosslinked polymer has taken up a large amount of a solvent and swollen, the interaction between polymer chains may be in a negligible state. However, in a solvent-free elastomer, the polymer chains forming the elastomer are tightly packed together, and thus viscosity is generated by the frictional force between the polymer chains. As a result, the elastomer has viscous properties in addition to the elastic properties exhibited by the crosslinked body as an assembly of chains exhibiting Hookean elasticity without contact with each other as described in the preceding section.

Depending on external conditions such as deformation speed, temperature, the molecular weight of polymer chains that make up the elastomer and the rigidity of the chains, whether the elastic properties or the viscous properties take precedence changes. Let us briefly explain these phenomena from the viewpoint of macromolecule theory[6].

When the molecular weight dependence of the viscosity of a polymer melt is examined, it is observed that the viscosity suddenly increases at a certain critical molecular weight. A state in which the molecular weight is higher than the critical molecular weight is called an ‘entangled state’, and a state in which it is lower is the ‘nonentangled state’. No transition is observed in the static properties, such as the molecular weight of the radius of gyration between the entangled state and the unentangled state. However, a transition phenomenon is observed for dynamic quantities such as viscosity. In the molecular theory of viscoelasticity of polymers, ‘entanglement’ refers to a constraint on the molecular motion caused by the fact that polymer chains cannot pass through each other because they are long string-like molecules. In the viscoelastic relaxation of a polymer melt that does not have a structure crosslinked by covalent bonds, the flat portion where the viscoelastic function does not depend on time or frequency is called a rubber-like region. In this region, the effect of a crosslinking point is considered to be caused by ‘entanglement’ between polymer chains. From the shear modulus *G* of the rubber-like region, the interentanglement molecular weight *M*_e_ of the network formed by the ‘entanglement’ can be calculated by the following equation:

(5) $$G=\frac{\rho RT}{M_{e}}$$

Here, *ρ, R*, and *T* are the density, gas constant, and absolute temperature of the polymer melt, respectively. The value of *M*_e_ depends on the type of polymer and is stored in a database[14].

Let us briefly explain the modeling of the phenomenon of entanglement with the concept of a tube model[15]. Focus on any one polymer chain in the polymer melt. Since the polymer chains are squeezed together in the melt, the polymer tends to move in the direction of the main chain, exhibiting anisotropic motion. The longer the chain length of the polymer chain is, the more pronounced its anisotropic mobility. In other words, in the tube model, the polymer chain can move freely in the main chain direction, but movement in the orthogonal direction is limited to a distance that does not affect the surrounding polymer chains. The movement of the polymer chains in the melt is regarded as the state of the polymer chains confined in the tube. This movement is similar to that of reptiles, so it is called ‘reptation’.

**Figure 6.**
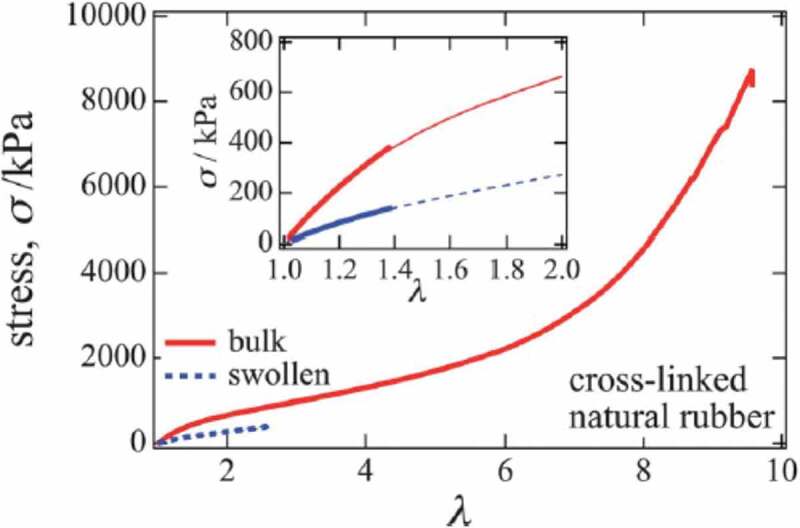
Stress–strain curves of crosslinked natural rubber and a gel in which the crosslinked natural rubber is swollen with a solvent (reproduced with permission from Elsevier)

Due to the viscous nature of such polymer chains in this tightly packed state, the elastomer is mechanically tougher than in the case of entropy elasticity alone. For example, it is clear from looking at the results of comparing the stress–strain curves of cross-linked raw rubber and gels obtained by swelling it with a solvent (Figure 6)[16]. Due to swelling, the breaking strength becomes 1/20, and the breaking strain becomes 1/3 or less. It is considered that the swelling of the crosslinked network with solvent significantly reduces the viscosity due to the friction between the polymer chains, resulting in deterioration of the mechanical properties of the gel.

Elastomers composed of amorphous polymers that exhibit viscous properties exhibit different stress behaviors during deformation loading and different stress routes during unloading, and this phenomenon is called hysteresis. A general elastic body stores energy by the application of strain, but since the stored energy is released in the absence of strain, no hysteresis is shown. In other words, it is known that the origin of hysteresis is the loss of energy due to the properties of the viscous material, and the presence of hysteresis increases the fracture energy of the elastomer. Grosch et al. have shown that hysteresis energy is involved in the fracture phenomenon of elastomers composed of amorphous polymers [17,18]. The relationship between the fracture energy *W* in the uniaxial extension of crosslinked rubber and the hysteresis energy *H* immediately before fracture is as follows:

(6) $$W=KH^{2/3}$$

Here, *K* is a constant.

**Figure 7.**
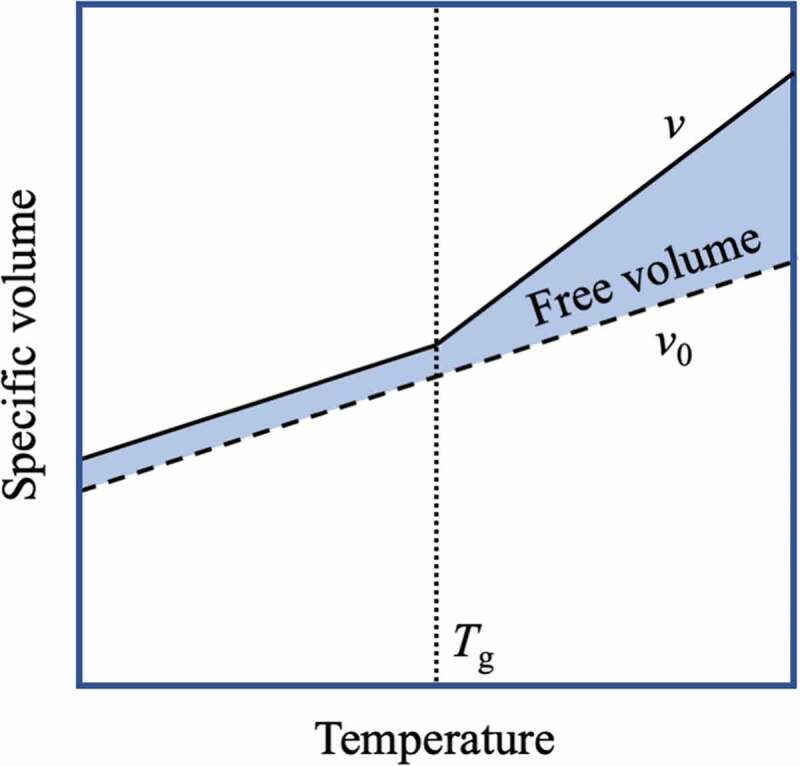
Temperature change in the free volume of an amorphous polymer

The amorphous polymer exhibits a transition from a state where the chain mobility is high to a state where the chain mobility is low as the temperature of the environment is lowered. This transition temperature is called the glass transition temperature (*T*_g_). At a sufficiently high temperature, rotation of the bonds of polymer chains occurs, and polymer chains move flexibly. However, at temperatures below *T*_g_, the motion of the polymer chains freezes completely. In other words, since the segment motion of the polymer chain changes greatly at *T*_g_, *T*_g_ can be obtained from the plot of the specific volume *v* of the polymer (reciprocal of the mass density of the polymer) against temperature. Simha and Boyer proposed that the volume of the polymer should be divided into the occupied volume *v*_0_ and the free volume *v*_f_ and that the general amorphous polymer becomes as shown in Figure 7 with *T*_g_ as the boundary[19]. The free volume is considered to be a void that allows molecules to undergo amplitude motion and swap their positions. In addition, Doolittle expressed the relationship between the viscosity *η* of the polymer melt and the free volume fraction (*v*_f_/*v*) by the following formula:[20].

(7) $$h=Aexp\;\left(v/v_{f}\right)$$

*A* is a constant depending on the substance.

That is, in the temperature range of *T*_g_ or higher, the lower the temperature is, the higher the viscosity of the polymer melt. Therefore, below *T*_g_, the melt shows the properties of an elastic body in which the motion of the molecular chain is restrained, but above *T*_g_, the energy dissipation increases as it approaches *T*_g_. The frictional force that causes the viscosity is essentially due to the molecular chains and van der Waals forces that attract the molecular chains. Therefore, the smaller the free volume, the higher the viscosity. From the above, it can be expected that an elastomer composed of an amorphous polymer exhibits viscous properties at a temperature of *T*_g_ or higher and exhibits high hysteresis and high toughness at temperatures close to *T*_g_.

### 5. Effects of introducing various bonds between polymer chains: introduction of reversible crosslinked structure

**Figure 8.**
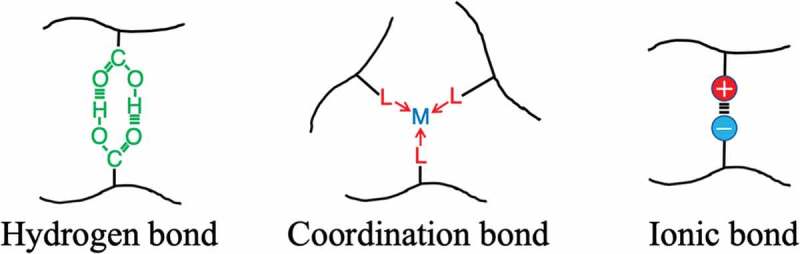
Schematic diagram showing reversible formation–dissociation bonds between polymer chains

**Figure 9.**
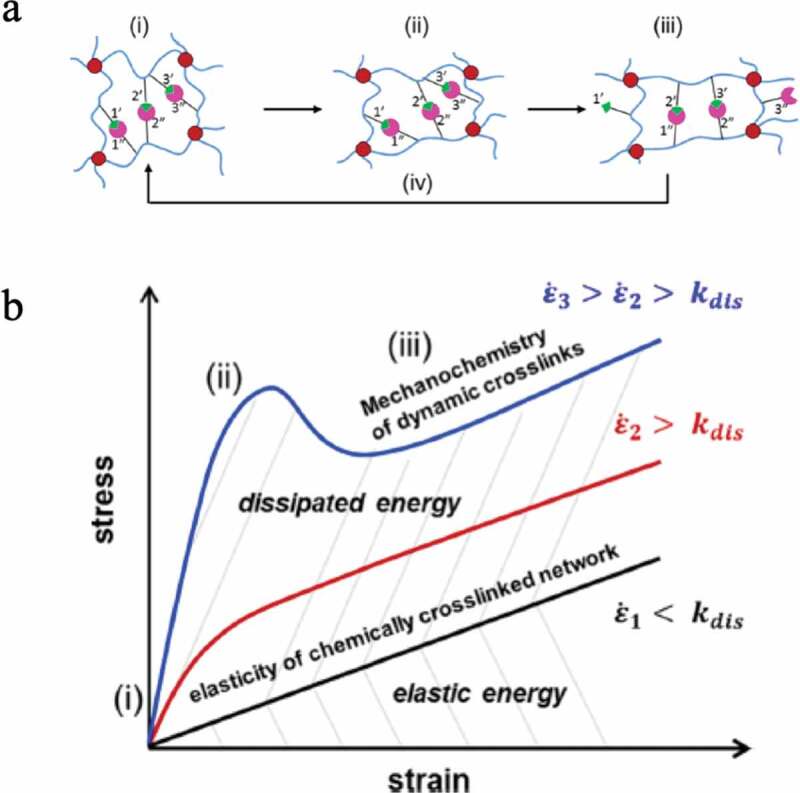
(a) Change in network structure when a three-dimensional crosslinked polymer consisting of a covalent crosslinking structure and reversible crosslinking structure is deformed. (b) Strain rate dependence of the stress–strain curve of a three-dimensional crosslinked polymer composed of a covalent crosslinking structure and reversible crosslinking structure (reproduced with permission from American Chemical Society)

As a recent strategy in the development of high toughness elastomers, in addition to the conventional crosslinked structure, the construction of a three-dimensional crosslinked polymer incorporating a reversible bond that can efficiently lose energy is being undertaken. Hydrogen bonds, coordination bonds, ionic bonds, etc. are adopted for the reversible bond, and attempts have been made to introduce a supramolecular structure that efficiently combines them (Figure 8). The conventional stable crosslinked structure has the role of maintaining the elastic properties and shape of the elastomer because the crosslinked structure does not change even when the elastomer is deformed. On the other hand, reversible bonds undergo repeated dissociation and formation of the crosslinked structure as the elastomer deforms (Figure 9(a))[21]. That is, when a reversible crosslinking structure is introduced, energy is dissipated by the deformation of the elastomer, which makes the elastomer highly tough. In addition, the stress–strain curve of the elastomer changes greatly depending on how fast the elongation (*ε*) is relative to the reversible bond dissociation rate (*k*_dis_) (Figure 9(b)). Even a gel swollen with a large amount of solvent is made tough by the introduction of a reversible bond. Furthermore, the synergistic effect of stable crosslinking and reversible crosslinking provides the elastomer with self-repairing properties and shape memory ability. Here are some examples.

**Figure 10.**
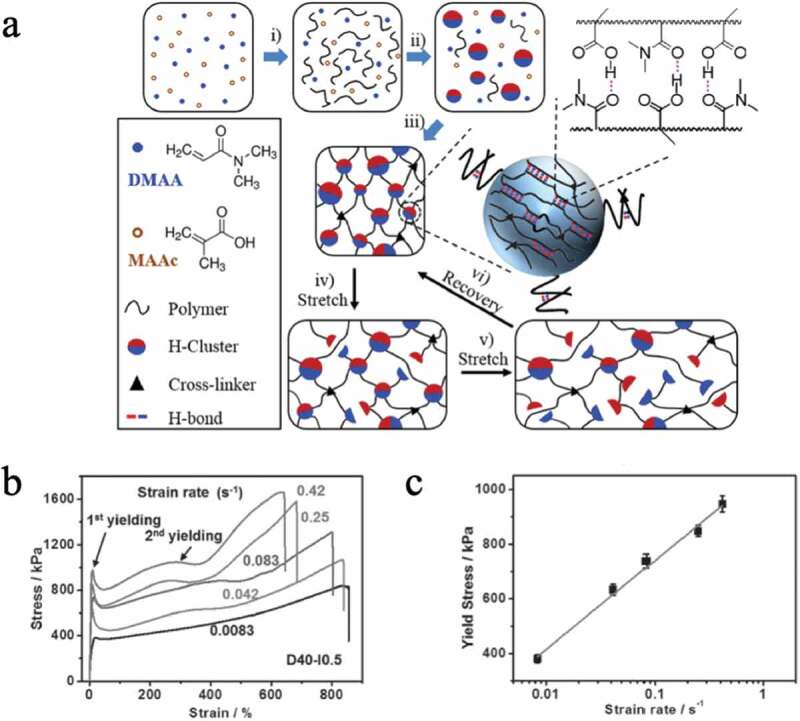
(a) A three-dimensional crosslinked polymer consisting of dimethyl acrylamide (DMAA), methacrylic acid (MAAc) and a crosslinking agent. Hydrogen bonds are formed between the polymers in the network. (b) Strain rate dependence of the stress–strain curve in (a). (c) Relationship between yield stress and strain rate obtained from the result in (b) (reproduced with permission from Wiley)

Sheiko et al. found that dimethylacrylamide and methacrylic acid, which are the monomers that make up the low-crosslinking three-dimensional polymer network, form hydrogen bonds within the network even in water, resulting in a high-strength hydrogel (Figure 10(a))[22]. A stress-strain test at a strain rate of 0.1 s^−1^ showed that the elastic modulus was 28 MPa, the fracture strength was 2 MPa, and the fracture energy was 9,300 Jm^−2^. It has also been confirmed that even if a large deformation of approximately 600% is applied, this hydrogel will return to its original state within 3 min or a few seconds at 37°C or 50°C, respectively. Furthermore, they found that the yield stress increases with increasing strain rate (Figure10(b and c)). This is a result showing that an elastomer with extremely high elasticity, high strength and high toughness compared with conventional hydrogels can be obtained by imparting hydrogen bond forming ability to the network.

**Figure 11.**
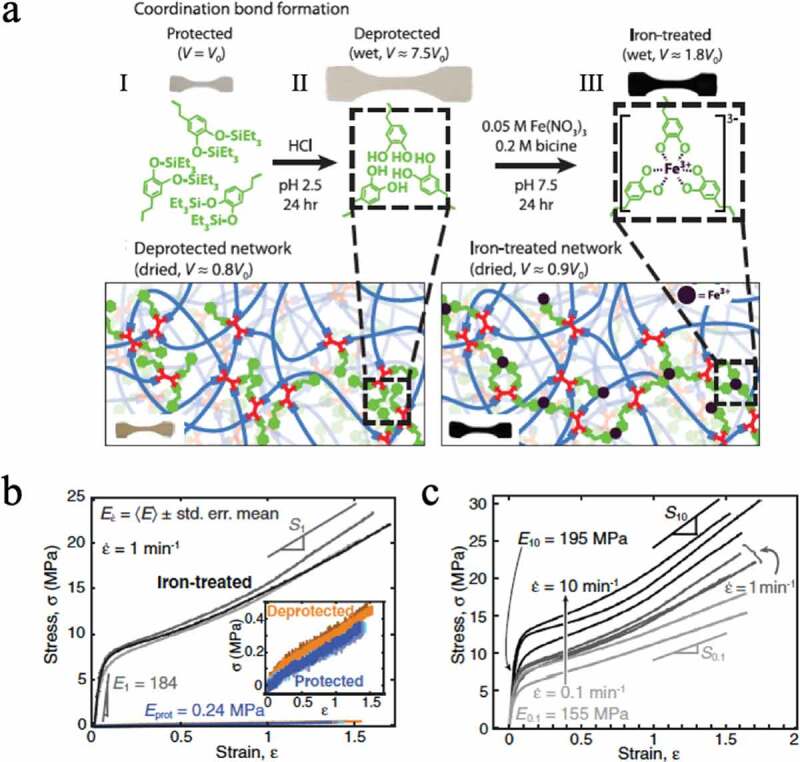
(a) Schematic diagram of a crosslinked polyethylene glycol chain in which a catechol group protected by (I) a silyl group, (II) a catechol group, and (III) a catechol group with a coordination bond to iron ion are introduced. (b) The stress–strain curve and strain rate of the elastomer in state III are 1 min^−1^. The inset shows (I) blue and (II) orange stress-strain curves with a strain rate of 1 min^−1^. (c) Strain rate dependence of stress–strain curve III (reproduced with permission from ASSS)

Filippidi et al. introduced iron-catechol crosslinks into a solvent-free three-dimensional network consisting of polyethylene glycol chains, referring to the high adhesiveness of the cuticle of mussel byssus[23]. In the state where the hydroxyl group of the precursor catechol group before the iron-catechol crosslinking is protected (Figure 11(a), I) and when the protective group is removed (Figure 11(a), II), the elastic modulus in the latter case is doubled according to the results of the stress-strain test of the elastomer, and the yield point occurs around the strain value *ε* of 0.2 (Figure 11(b) inset). This is believed to be due to the presence of weak reversible hydrogen bonds between the catechol groups. Furthermore, when iron ions are added to an elastomer with catechol groups, the catechol groups form iron-catechol bridges with iron ions (Figure 11(a), III). The iron-catechol bridges have much higher dissociation energies than hydrogen bonds between catechol groups, resulting in a 770-fold elastic modulus and a 58-fold fracture strength compared to the iron-free precursor (Figure 11(b)). Additionally, the yield stress increases with increasing strain rate (Figure 11(c)). Recoverable hysteresis energy dissipation was obtained, and the breaking energy was increased 92 times. Studies on iron-catechol crosslinks have been conducted in hydrogels, but in solvent-free elastomers, iron-catechol crosslinks are more effective for increasing the toughness of elastomers.

**Figure 12.**
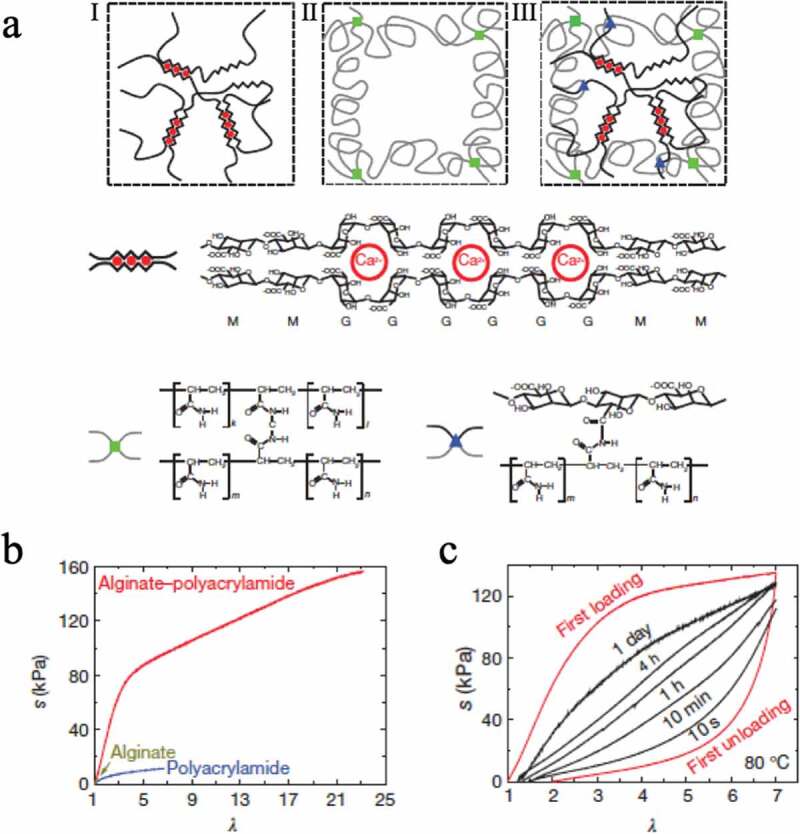
(a) Schematic representation of three different networks: (I) network formed from alginate and calcium ions, (II) covalently crosslinked polyacrylamide network, and (III) a combination of networks I and II. (b) Stress–strain curves of gels composed of the three different networks. (c) The stress–strain curve after uniaxially stretching the gel with network III and holding it at 80°C for a certain time shows the recovery of the sacrificial bond with time (reproduced with permission from Nature Publishing Group)

Suo et al. prepared a gel (Figure 12(a), III) that combines a reversible ionic bond crosslinking structure (Figure 12(a), I) and a covalent crosslinking structure (Figure 12(a), II)[24]. The ionic bond utilizes the interaction between alginic acid and calcium ions. Furthermore, alginic acid is covalently bound to the low crosslinking structure of acrylamide. In this system, it was also reported that the former is responsible for energy loss and that the latter maintains the memory of the network structure, resulting in a hydrogel with extremely high toughness (Figure 12(b)) and high recovery ability (Figure 12(c)).

**Figure 13.**
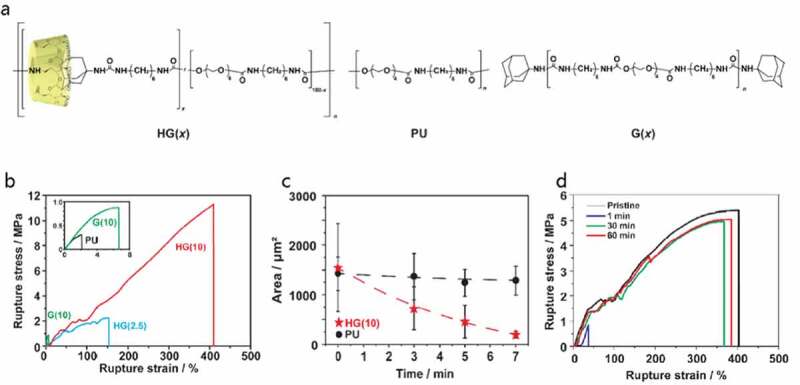
(a) Structures of thermoplastic polyurethane linked with host–guest interactions (HG), linear polyurethane (PU) and polyurethane with only the guest monomer (G) are compared. Here, (x) indicates the mol% of the host and guest units. (b) Stress–strain curve of the prepared gel. (c) Relationship between time and damaged area, which represents the self-healing ratio. (d) Stress–strain curve of cut HG_(10)_ after recovering for 1, 30, 60 min and original HG_(10)_, which represents the self-healing ratio by calculating the fracture energy (reproduced with permission from MDPI)

Takashima et al. prepared a thermoplastic gel (Figure 13(a)) by introducing host-guest interactions, which improved its mechanical properties and self-healing ability[25]. In this system, host-guest interaction between permethylated amino β-CD (PMeAmβ-CD) and adamantane amine (AdAm) was introduced into thermoplastic polyurethane (TPU). In this study, TPU gel linked with 10 mol% host-guest interaction had the highest fracture strength and fracture energy of 11 MPa and 25 MJm^−3^ (Figure 13(b)), almost 40-fold and 1500-fold higher than those of nonfunctionalized TPU (TEG-based), respectively. Furthermore, this gel has 87% mechanical properties after 7 min of recovery at 80°C from damage (Figure 13(c)) and 80% mechanical properties after 60 min of recovery at 80°C from fracture (Figure 13(d)). Combining such host-guest interactions with covalently bonded crosslinked structures would be very effective in developing tough elastomers. It is also very useful to introduce the concept of the vitrimer proposed by Leibler et al. into elastomers[26].

These systems can increase the toughness of an elastomer by utilizing a sacrificial bond whose dissociation enables energy dissipation during deformation of the elastomer [27–40]. This study seeks to increase the toughness of elastomers by utilizing energy dissipation due to the interaction between molecules, compared with the van der Waals force explained in the previous section. A system has also been proposed in which two different network structures form a mutually interpenetrating structure so that the covalent bonds of one network are used as sacrificial bonds, and the other network is used for structural maintenance[41].

### 6. Soft material with crosslinked structure that can move freely

**Figure 14.**
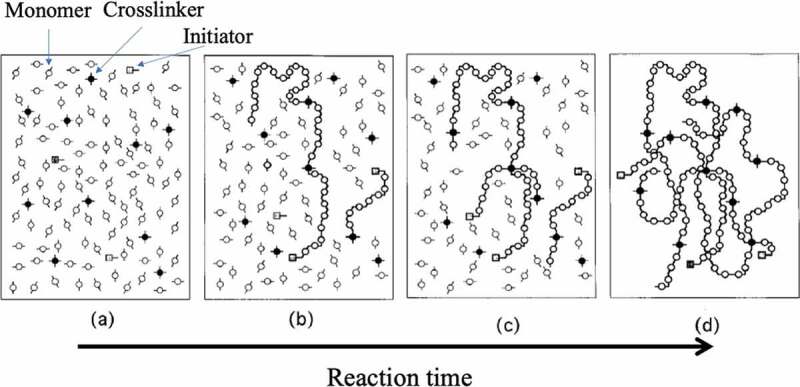
Conceptual diagram of the formation process of the network structure by general free radical polymerization (reproduced with permission from American Physical Society)

**Figure 15.**
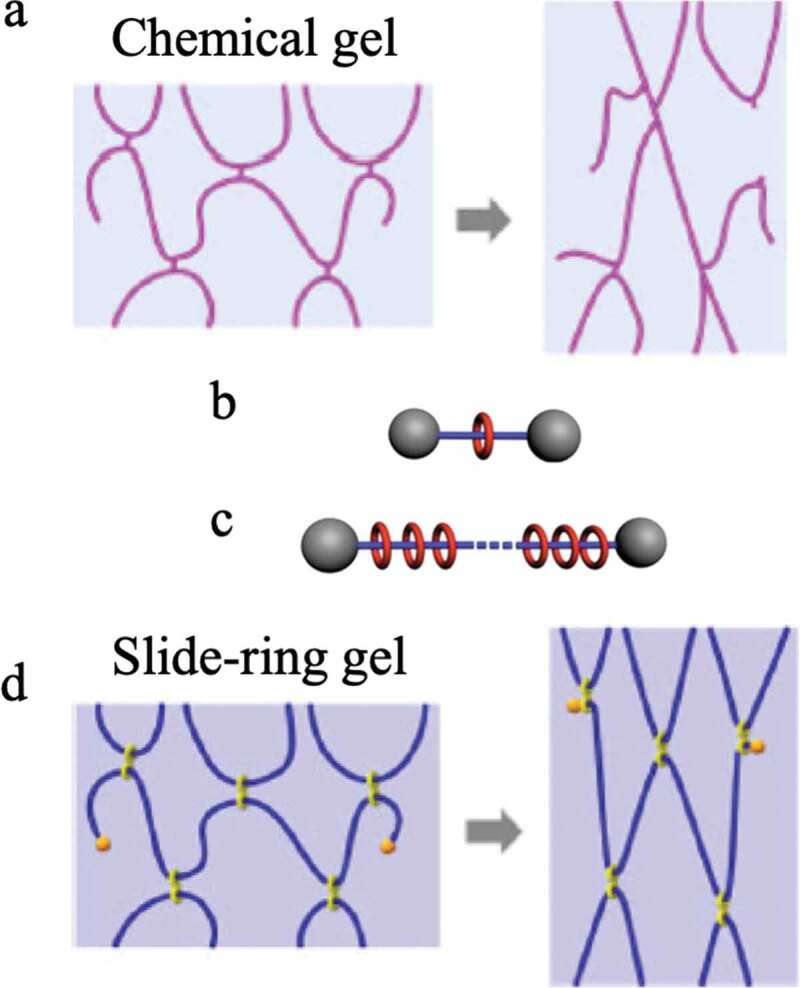
(a) Conceptual diagram showing bond cleavage due to strain applied to a chemically bonded three-dimensional crosslinked network in which the length of the polymer chains between the crosslinking points is nonuniform (reproduced with permission from Nature Publishing Group.), (b) Schematic diagram of rotaxane. (c) Schematic diagram of polyrotaxane. (d) Change in network structure when strain is applied to the slide-ring gel obtained by crosslinking cyclic molecules between polyrotaxanes

When a three-dimensional network with crosslinked polymer chains is synthesized by a random reaction between a monomer and a crosslinking agent, judging from the general reaction mechanism, the length and the number of polymer chains existing between the crosslinking points of the obtained three-dimensional network, the size and shape of the network are not constant (Figure 14)[42]. That is, the network structure of the elastomer that we have used so far does not consist of uniform length polymer chains between the crosslinking points, as shown in Figure 4(b). Therefore, when an external force is applied to deform an elastomer with a general chemical crosslinking structure, stress concentrates on the portion where the chain length between the cross-linking points is the shortest (Figure 15(a)). As a result, the elastomer breaks mechanically. Ito et al. succeeded in avoiding the concentration of stress on some chains during the deformation of the elastomer by introducing into the elastomer a molecular structure that allows the crosslinked portion to move freely [4,43]. The molecule used for that purpose is a polymer with a special structure called polyrotaxane (PR). Molecules that have a structure in which an axial molecule penetrates a cyclic molecule are called rotaxanes, and a bulky site is attached to both ends of the axial molecule so that the cyclic molecule does not escape (Figure 15(b)).In PR, a polymer chain is used as the axial molecule, and a large number of cyclic molecules can be included (Figure 15(c)). Reducing the number of cyclic molecules in PR can allow the cyclic molecules to move within PR. The best-known system among PRs is a PR synthesized by Harada et al. from linear polyethylene glycol (PEG) and cyclic α-cyclodextrin (α-CD) [44,45]. The rotaxane structure is formed simply by mixing solutions of PEG and α-CD in water, and a stable PR can be obtained by modifying both ends of PEG at bulky sites such as adamantane. Ito et al. succeeded in synthesizing gels with extremely flexible mechanical properties by crosslinking the α-CDs of PR (Figure 15(d)). The crosslinking sites generated by the bonding of the cyclic molecules produce a state in which it can move freely in the network. As a result, when the gel is deformed, the tension of the polymer chains moves to adopt a uniform state, and the nonuniformity of stress can be dispersed. Therefore, this PR gel exhibits mechanical flexibility.

Takeoka et al. have been working on the use of PR as a crosslinking agent for application to various elastomers [46–51]. Since the α-CD in PR has a large number of reactive hydroxyl groups, modifying the vinyl groups that can react with various vinyl monomers makes it possible to easily prepare a three-dimensional network composed of various polymers. By synthesizing an elastomer using such a PR crosslinking agent (PR crosslinking agent), it is possible to create a supramolecular network in which the crosslinking points can move freely over the elastomer obtained using conventional crosslinking agents. If the respective polymer chains in the elastomer become fully extended due to the movement of the crosslinking points, it may be possible to sufficiently exhibit the elongation of the polymer chains. Furthermore, in the case of an elastomer containing no solvent, if the fluidity of the polymer chains constituting the elastomer is increased, the energy generated by the deformation of the elastomer due to the force applied from the outside is dissipated, and therefore improved toughness can be expected.

This section will introduce the mechanical properties of an elastomer synthesized from this PR crosslinking agent and a methacrylate monomer.

**Figure 16.**
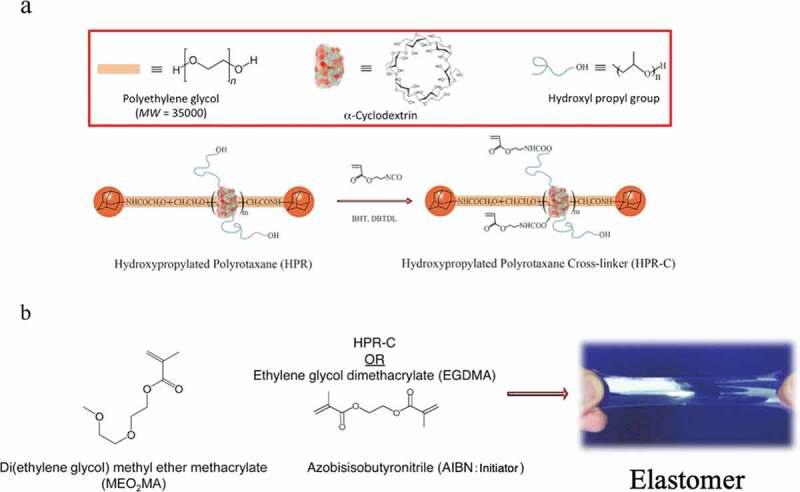
(a) Schematic diagram of polyrotaxane and polyrotaxane crosslinkers formed from polyethylene glycol and α-CD. This polyrotaxane has α -CD modified with a hydroxypropyl group to improve its solvent affinity. (b) Elastomer obtained by using MEO_2_MA as a monomer in combination with the polyrotaxane cross-linking agent or a general cross-linking agent EGDMA (reproduced with permission from ASSS)

A polyrotaxane crosslinking agent (HPR-C) (Figure 16(a)) obtained from a derivative (HPR) synthesized by adding a hydroxypropyl chain to the α-CD of PR was used. Furthermore, diethylene glycol methyl ether methacrylate (MEO_2_MA) (Figure 16(b)), which is in a liquid state at room temperature for both the monomer and the polymer, was used as the monomer. MEO_2_MA and HPR-C were dissolved in dimethylsulfoxide (DMSO) together with the radical initiator azobisisobutyronitrile (AIBN), and a gel swollen with DMSO was obtained by thermal polymerization. Then, unreacted materials and DMSO were washed with methanol, and methanol was distilled off to obtain an elastomer composed of MEO_2_MA and HPR-C (Figure 16(b)). For comparison, an elastomer composed of MEO_2_MA using ethylene glycol methyl ether methacrylate (EGDMA) as a typical cross-linking agent was also synthesized (Figure 16(b)).

**Figure 17.**
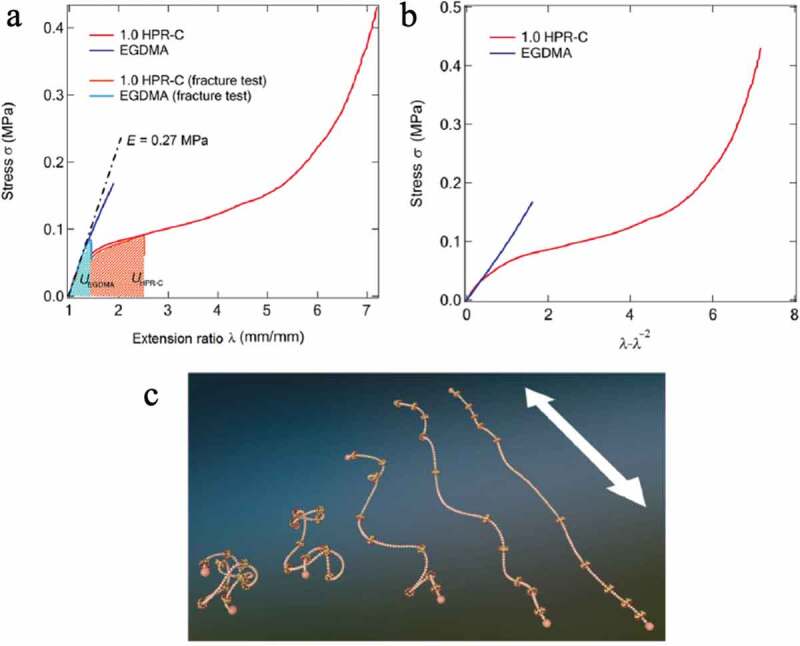
(a) Stress–strain curves of two elastomers with different crosslinking agents shown in Figure 16b, (b) the figure in which the horizontal axis in a) is changed to *λ *− *λ*^−2^. (c) Conceptual diagram showing changes in polyrotaxane conformation based on SAXS results observed during uniaxial stretching of an elastomer prepared using the polyrotaxane crosslinking agent (reproduced with permission from ASSS)

Figure 17(a) shows the result of a stress-strain test in which a dumbbell-shaped sample was prepared from an elastomer prepared from HPR-C and MEO_2_MA and a MEO_2_MA elastomer using EGDMA as a cross-linking agent and subjected to uniaxial stretching. The Young’s modulus *E* obtained from the slope of the initial proportional portion of the stress–strain curve is approximately 0.27 MPa for both elastomers. The relationship between stress and strain in uniaxial stretching can be rewritten as follows, assuming that the polymer chain that constitutes the elastomer is a Gaussian chain:

(8) $$\sigma =\frac{\rho RT}{M_{c}}\left(\lambda -\frac{1}{\lambda^{2}}\right)$$

Here, *ρ* is the material density, *R* is the ideal gas constant, *T* is the temperature, and *M*_c_ is the average molecular weight between the crosslinking points of the polymer chains constituting the elastomer. Figure 17(b) shows the results of plotting stress σ against *λ *− *λ^−^*^２^ based on the results of the stress-strain test of both elastomers in Figure 17(a). Since the system using EGDMA has a linear relationship with a constant slope, it behaves in accordance with the relationship of the above equation (8). The slope *ρRT*/*M*_c_ = *G* = 0.1 MPa, and *G* is the shear elastic modulus. Assuming Poisson’s ratio *ν *= 0.5, Young’s modulus becomes *E* = 2 *G* (1 + *ν*) = 3 *G*, which is almost the same as Young’s modulus *E* = 0.27 MPa obtained from the stress–strain curve. Therefore, it is understood that the stress-strain behavior of the elastomer using EGDMA is in accordance with Gaussian theory.

On the other hand, the elastomers using HPR-C behaved completely differently, and Gaussian theory could not be applied, suggesting that they have different crosslinked network structures. In addition, the strain value at break *ε* was 75% for the system using EGDMA and 620% for the system using HPR-C.

From SAXS measurement with uniaxial elongation of the elastomer using HPR-C, it has been confirmed that the crosslinking agent HPR-C also has a conformation elongated in the elongation direction of the elastomer, and α-CD is also arranged in the elongation direction of PR (Figure 17(c)). Although the behavior of the polymer chains constituting the elastomer has not been confirmed, it is considered that each polymer chain also expands greatly according to the elongation of the crosslinking agent and the movement of α-CD. Systems combining such migrating crosslinkers with reversible bonds are also of interest.

## 7. Conclusions and future perspectives

This paper first describes the classical explanation of the mechanical properties of elastomers based on the handling of single-chain extension. Large deformation, however, was not possible to explain with classical theory, and it was proposed that the experimental result may stem from behavior that is significantly different from the explanation provided classical theory. Additionally, in an actual elastomer, it is necessary to take into account interactions and entanglements between molecules, and the presence of interactions between polymer chains allows energy dissipation to increase the toughness of the elastomer. Taking this into consideration, the elastomer can be made tougher by introducing strong intermolecular interactions. Furthermore, since it has become possible to develop elastomers with moving crosslinking points, the combination of reversible formation–dissociation interactions and moving crosslinking points may lead to elastomers with better elongation and higher toughness.
